# Haematological changes in *Schistosoma haematobium* infections in school children in Gabon

**DOI:** 10.1007/s15010-020-01575-5

**Published:** 2021-01-24

**Authors:** Jean Claude Dejon-Agobé, Ayôla A. Adegnika, Martin P. Grobusch

**Affiliations:** 1 Centre de Recherches Médicales de Lambaréné (CERMEL), Lambaréné, BP 242, Lambaréné, Gabon; 2 Center of Tropical Medicine and Travel Medicine, Department of Infectious Diseases, Division of Internal Medicine, Amsterdam University Medical Centers, Location AMC, Amsterdam Public Health, Amsterdam Infection and Immunity, University of Amsterdam, Amsterdam, The Netherlands; 3 Department of Parasitology, Leiden University Medical Center, 2333 ZA Leiden, The Netherlands; 4 Institut Für Tropenmedizin, Eberhard Karls University Tübingen, 72074 Tübingen, Germany; 5 German Center for Infection Research, Partner Site Tübingen, 72074 Tübingen, Germany; 6 Fondation Pour la Recherche Scientifique, 72 BP 045 Cotonou, Benin; 7 Masanga Medical Research Unit (MMRU), Masanga, Sierra Leone; 8 Institute of Infectious Diseases and Molecular Medicine (IIDMM), Cape Town, Republic of South Africa

**Keywords:** Schistosomiasis, *Schistosoma haematobium*, Haematology, School children, Gabon

## Abstract

**Background:**

Schistosomiasis is a parasitic disease affecting the blood cell. As a chronic disease, schistosomiasis particularly impacts on the human host’s haematological profile. We assessed here the impact of urogenital schistosomiasis on the full blood counts (FBC) as proxy diagnostic tool for schistosomiasis.

**Methods:**

A cross-sectional study was conducted among school children living in Lambaréné, Gabon. Schistosomiasis status was determined using urine filtration technique. EDTA blood samples were analysed using a Pentra ABX 60^®^ analyzer.

**Results:**

Compared to their infection-free counterparts, school children infected with *Schistosoma haematobium* displayed an altered FBC profile, with changes in all three blood cell lines. Adjusted for praziquantel intake, soil-transmitted helminthic infections and *Plasmodium falciparum* infection status, schistosomiasis was independently associated with a decreasing trend of mean haemoglobin (β = − 0.20 g/dL, *p-value* = 0.08) and hematocrit (β = − 0.61%, *p-value* = 0.06) levels, a lower mean MCV (β = − 1.50µm^3^, *p-value* = 0.02) and MCH (β = − 0.54 pg, *p-value* = 0.04), and higher platelet (β = 28.2 10^3^/mm^3^, *p-value* = 0.002) and leukocyte (β = 1.13 10^3^/mm^3^, *p-value* = 0.0003) counts, respectively.

**Conclusions:**

Schistosomiasis is associated with a characteristic FBC profile of schoolchildren living in Lambaréné, indicating the necessity to consider schistosomiasis as a single cause of disease, or a co-morbidity, when interpreting FBC in endemic areas.

## Introduction

Schistosomiasis is one of the two most-common parasitic infections globally, with transmission reported from 78 countries [1]. Globally, 700 million people live in endemic areas [2]. Among the now 240 million people estimated of having schistosomiasis and requiring treatment, more than 90% are estimated to live in sub-Saharan Africa [2, 3].

Schistosomiasis leads both to acute and chronic disease. At both stages, schistosomiasis stimulates the host immune system, being in part reflected in routinely measurable biochemical and, in focus here, haematological alterations. In general, higher mean leukocyte counts and changes in mean of differential leukocyte counts are observed among individuals infected with schistosomiasis compared to those without; both normalise following praziquantel (PZQ) treatment [4]. Eosinophilia is characteristic for schistosomiasis [5]. Thrombocytes play a protective role against schistosomiasis by exerting direct damaging effects on adult worms, with platelet count changes depending on the disease phase [6]. In mice, thrombocytes were shown to adhere to the surfaces of, and to kill mechanically transformed schistosomula, leading to thrombocytopenia in early disease [7]. In the brown rat, a three–fourfold increase of thrombocytes protective against schistosomes was observed 4–6 weeks after initial infection [8]. Few studies were conducted to assess thrombocyte changes in humans [9, 10], and the potential anthelminthic effects thereof. One study reported a thrombocyte reduction by half in patients with intestinal schistosomiasis. However, the authors concluded that this was more likely attributable to portal hypertension rather than directly to the helminths [9]. Another study reported a trend for thrombophilia among Ghanaian children with urogenital schistosomiasis compared to their non-infected counterparts [10]. In addition to leucocytes and platelets, the third blood cell line does not remain unaffected by schistosomiasis either. The main symptom of schistosomiasis is the presence of blood in urine (in urogenital schistosomiasis) [11] or in stool (in intestinal schistosomiasis), due to blood vessel rupture during egg excretion. Chronic schistosomiasis, which particularly affects school-aged children, adolescents and young adults has been reported to be associated with chronic inflammation and iron deficiency anaemia [12].

With schistosomiasis being mainly a chronic infection and very often asymptomatic at that stage, and knowing that schistosomiasis is able to influence the mean FBC parameters as discussed above, we can, thus, assume that in case of co-infections with other diseases such as malaria, schistosomiasis might eventually go unnoticed if the clinician is unaware of the presence of this (co)morbidity. Indeed, in malaria-negative individuals in co-endemic areas, for example, a certain level of microcytic anaemia on its own would probably go unnoticed as it is explained by repeated malaria episodes, with anaemia being one of the malaria-diagnostic criteria for disease severity, and chronic anaemia being a well-recognised consequence of repeated malaria episodes. A recent epidemiological assessment demonstrated that Lambaréné is an area of urogenital schistosomiasis with a 26% prevalence, with hematuria and proteinuria being positively associated with the disease [13]. In this follow-up analysis, we examined the effect of schistosomiasis on FBCs as a surrogate diagnostic parameter, among school children living in Lambaréné, an area endemic for *S. haematobium* and where PZQ is available.

## Materials and methods

### Study area

Lambaréné is a town in Gabon located 80 km south of the equator known to be endemic for schistosomiasis. The predominant *Schistosoma* species is *S. haematobium* [13–16]. Lambaréné is also known to be endemic for soil-transmitted helminths (STH) [17] and malaria, with highest prevalences in school children and adolescents [18].

### Study design and population

The study design was cross-sectional. Volunteers were recruited amongst consenting eligible, apparently healthy school children living in Lambaréné.

### Sample size consideration

The current sample size was calculated for a cross-sectional study aiming to determine the prevalence of schistosomiasis in Lambaréné and associated factors, as described elsewhere [13]. In the current analysis, we assessed the difference in haematological parameters between participants with schistosomiasis and those without. Considering a 5% type-I error, and having 161 participants included in the Schistosoma-positive group and 451 in the control group, we were able, with more than 90% power, to detect a minimum of 10% between both groups for platelet levels, and more than 10% for WBC and RBC levels.

### Study procedures and laboratory examinations

The study was conducted from April to July 2016. Participants were selected at school as described elsewhere [13]. Briefly, legal representatives of volunteers invited at school to partake were visited at home and asked to grant informed consent. Trained field workers used a standardised questionnaire to inquire with parents or other primary caretakers about, among other, history of passing blood in urine and treatment received (PZQ or other anthelminthic drugs) in the previous six months. Nurses collected study subjects’ demographic data (age, sex and address) at school. In cases of acute medical concerns, the participant was referred to the clinician for appropriate care.

Eligible participants were provided with plastic containers at school and were invited to provide three urine samples on three consecutive days, and one stool sample at earliest convenience. For each urine sample, urine filtration was performed for the detection of *S. haematobium* eggs using a Whatman microfilter membrane of 10–12 µm as described elsewhere [19]. We used the Kato-Katz technique for the detection of eggs of STH in stool samples as described elsewhere [20] and the copro-culture technique for the detection of hookworm (*Necator americanus*) larvae [13, 20]. One 2.7 mL blood sample was collected into an ethylenediaminetetraacetic acid (EDTA) tube to perform a FBC, using the automated haematology analyser (ABX Pentra 60^®^, Horiba Instruments Incorp., Irvine, CA < USA). In addition, 10 μL of fingertip blood was collected for a thick blood smear (TBS) for the detection of *Plasmodium* spp. parasites applying the Lambaréné method [21]. A rapid diagnostic test (RDT) was performed for the rapid detection of circulating *P. falciparum* HRP-2 antigen using the Paracheck *Pf*^®^ Malaria Test (Orchid Biomedical Systems, Goa, India).

Participants found positive for *Schistosoma* eggs were treated with 40 mg/kg of PZQ once. Those testing STH- or Plasmodium positive were treated with oral albendazole 400 mg daily for three consecutive days [22], or with artemisinin-based combination therapy (ACT) [23], respectively. For other infections, medical prescription was given by the study clinician, or the participant was referred to an appropriate health centre.

### Statistical considerations

We collected data using the patient report form and digitalised it, using the REDCap electronic data capture tool [24] hosted at CERMEL. The original clean database was exported into R software (Version 3.2.4) and a subset database (see Supplementary Database) was obtained for the statistical analyses. Age was used as categorical variable, grouped in 5-year strata. Participants were considered having malaria when testing positive either with TBS or RDT. Qualitative variables were summarised by proportion and 95% confidence interval (CI); while, quantitative variables were summarised by mean and standard deviation (SD) or by median and interquartile (IQ) range where appropriate. The normality of the distribution of continuous variables was assessed by visual inspection and if needed, a log transformation was performed. Chi-square test was used to compare qualitative variables. Student’s *t*-test was used to compare means of continuous variables; while, the Wilcoxon test was used to compare their distribution. Linear Model (LM) regression was used to correct for confounding factors potentially influencing haematological parameters with regard to *Schistosoma* status. The residuals were used to check for assumptions to ensure the usefulness of the model. The level of statistical significance was set at less than 0.05.

### Ethical considerations

The original study protocol was approved by the institutional ethic committee of CERMEL (CEI-CERMEL 002/2016). The study was conducted in line with the Good Clinical Practice (GCP) principles of the International Conference on Harmonization (ICH) [25] and the Declaration of Helsinki [26].

## Results

### Study population characteristics

A total of 614 participants were included in the original study [13], and 612 of them with haematology data available were incorporated in this analysis. The mean age of our study population was 10.1 (SD = 2.7) years, with a 0.95 female:male ratio. The mean haemoglobin level was 11.6 (SD = 1.13) g/dL. Ten per cent [_95%_CI: 8–13] and 23% [_95%_CI: 19–26] of our study population, respectively, declared having taken PZQ or albendazole treatment within the past six months prior to study enrolment. The prevalence of *Plasmodium* spp. infection combining TBS and RDT was 20% [_95%_CI: 17–23]. The prevalence of any STH was 15% [_95%_CI: 12–18].

### Study group characteristics

The 161 (26%) participants found schistosoma-egg positive (159 in urine and two in faeces) constituted the schistosoma positive (Sch +) group; while their negative counterparts constituted the schistosoma-negative (Sch-) group, leading to a ratio of 2.8 non-infected participants per single infected participant (Table 1). The two groups were comparable for age (*p-value* = 0.33) and sex (*p-value* = 0.39). Of note, the proportion of the participants previously treated with PZQ was significantly higher among the Sch + group compared to the Sch- group (16% vs 8%, *p-value* = 0.008). The overall prevalence of STH was higher among the Sch + group compared to the Sch- group (22% vs 12%, *p-value* = 0.005). Trichuriasis was the most prevalent STH infection in both groups, and was mostly prevalent among the Sch + group compared to the Sch- group (17% vs 8%, *p-value* = 0.001). Plasmodium infection was more prevalent in Sch + group than Sch- group (28% vs 17%, *p-value* = 0.003), particularly when detected using RDT.

**Table 1:** Characteristics of the 612 study participants with regard to schistosomiasis infection status

			Overall study population		Schistosoma negative		Schistosoma positive		*p* value
			*N*	%, 95% CI	*N*	(%)	*n*	(%)	
Sample size			612	–	451	(73.7)	161	(26.3)	
Sex									**0.39**
	Female		299	48.9 [40.2–48.2]	225	(49.9)	74	(46.0)	
	Male		313	51.1 [40.7–55.2]	226	(50.1)	87	(54.0)	
Sex ratio (Female/Male)			**0.95**		**1.00**		**0.85**		
Age (mean, sd)			**10.1**	**2.7**	**10.1**	**2.7**	**10.0**	**2.8**	**0.83**
Age									**0.33**
	5–9		352	57.5 [53.5–61.5]	262	(58.1)	90	(55.9)	
	10–14		239	39.1 [35.2–43.0]	171	(37.9)	68	(42.5)	
	15–19		21	3.4 [2.1–5.2]	18	(4.0)	3	(1.9)	
*Plasmodium* infection*									
	By microscopy		36	5.9 [4.2–8.1]	23	(5.1)	13	(8.2)	0.16
		Parasitemia (Geomet-ric mean)		756		574		1229	0.28
	By RDT		118	19.3 [16.3–22.7]	75	(16.7)	43	(26.7)	0.006
	Any method		122	20.1 [17.0–23.5]	77	(17.2)	45	(28.3)	0.003
History of praziquantel treatment**									**0.008**
	Yes		62	10.1 [7.9–12.8]	37	(8.3)	25	(15.7)	
STH***									
	Ascariasis		15	3.2 [1.8–5.2]	7	(2.1)	8	(6.1)	0.03
	Trichuriasis		49	10.4 [7.8–13.5]	26	(7.6)	23	(17.4)	0.001
	Hookworm		18	3.8 [2.3–6.0]	12	(3.5)	6	(4.5)	0.60
	Any STH		69	14.6 [11.6–18.1]	40	(11.8)	29	(22.0)	0.005
History of STH treatment****									**0.12**
	Yes		137	22.6 [19.3–26.1]	108	(24.2)	29	(18.1)	

^$^1 missing data

*5 missing data; 4 for microscopy examination, 2 for rapid diagnosis test including 1 for both

**Taken in the last 6 months, 7 missing data

***140 missing data; 111 for Schistosoma-negative group and 29 for Schistosoma-positive group

****5 missing data

### Haematological profile and schistosomiasis status

We assessed the haematological profile of the study participants according to schistosomiasis status (Table 2) in the absence of clinical signs or symptoms hinting at a possible concomitant infectious or non-infectious disease. We found significant lower haemoglobin levels among the schistosoma-positive participants compared to their schistosoma-negative counterparts (median: 11.5 g/dL vs 11.7 g/dL, *p-value* = 0.001). However, WBC (7.5 10^3^/mm^3^ vs 6.5 10^3^/mm^3^, *p-value* < 0.001) and thrombocyte (254 10^3^/mm^3^ vs 232 10^3^/mm^3^, *p-value* = 0.002) counts were significantly higher in children with schistosomiasis. Looking specifically at the differential leukocyte count, we found a higher level of lymphocytes (3.57 10^3^/mm^3^ vs 3.24 10^3^/mm^3^, *p-value* < 0.0001), neutrophils (2.49 10^3^/mm^3^ vs 2.29 10^3^/mm^3^, *p-value* = 0.01), eosinophils (0.52 10^3^/mm^3^ vs 0.30 10^3^/mm^3^, *p-value* < 10^–4^) and basophils (0.07 10^3^/mm^3^ vs 0.05 10^3^/mm^3^, *p-value* < 0.001) in Sch + compared to Sch-.

**Table 2:** FBC profile of the 612 study participants regarding schistosomiasis status

FBC parameters		Schistosoma status (median, [IQ])		*p* value (Wilcoxon test)
		Negative	Positive	
Erythrocytes (10^6^/mm^3^)		**4.56 [4.29–4.83]**	**4.54, [4.30–4.85]**	**0.88**
	Haemoglobin (g/dl)	11.7 [11.1–12.3]	11.5, [10.7–12.1]	0.001
	Hematocrit (%)	35.9 [34.2–35.8]	35.4, [32.7–36.6]	0.0005
	MCV (µm^3^)	79.0 [75.0–83.0]	77.0, [73.0–81.0]	< 0.0001
	MCH (pg)	25.9 [24.4–27.5]	25.1, [23.7–26.5]	< 0.0001
	MCHC (g/dl)	32.7 [32.0–33.2]	32.5, [31.8–33.2]	0.26
Thrombocytes (10^3^/mm^3^)		**232 [170–287]**	**254, [195–315]**	**0.003**
Leukocytes (10^3^/mm^3^)		**6.50 [5.50–8.00]**	**7.5, [6.30–9.10]**	** < 0.0001**
	Lymphocytes (10^3^/mm^3^)	3.24 [2.64–4.40]	3.57, [3.11–4.40]	< 0.0001
	Neutrophils (10^3^/mm^3^)	2.29 [1.79–3.09]	2.49, [2.04–3.19]	0.01
	Eosinophils (10^3^/mm^3^)	0.30 [0.16–0.50]	0.52, [0.33–0.90]	< 0.0001
	Basophils (10^3^/mm^3^)	0.05 [0.04–0.07]	0.07, [0.05–0.09]	< 0.0001
	Monocytes (10^3^/mm^3^)	0.39 [0.03–0.61]	0.43, [0.01–0.61]	0.50

A multivariate analysis was carried out to investigate a relationship between haematological constants and schistosomiasis status adjusted for PZQ intake, STH and *P. falciparum* infection status (Table 3), yielding a statistically significant positive correlation between thrombophilia and a diagnosis of schistosomiasis. Similarly, a statistically significant positive correlation between leukocyte count and a diagnosis of schistosomiasis was observed. This applied to all WBC subclasses except for monocytes, too (Table 3). For RBC, a relationship trend was found towards lower haemoglobin and haematocrit levels, respectively (Table 3). With regard to the corpuscular constants of RBCs, a significant relationship was observed for MCV (*p-value* = 0.02) and MCH (*p-value* = 0.04), not for MCHC (*p-value* = 0.83). Indeed, a decrease of 1.50 µm^3^ and 0.54 pg was observed in MCV and MCH mean level of participants with schistosomiasis, respectively. The adjusted R^2^ value for each haematological constant was basically less than 0.01; so, less than 10% of the variation in these constants can be explained by our regression model. The data met the assumptions of homogeneity of variance and linearity and the residuals were approximately normally distributed.

**Table 3:** Differences in mean of the FBC parameters with regard to schistosomiasis status and adjusted on *Plasmodium* infection, any STH infection status, and history of praziquantel intake among the 472 participants with completed results

FBC parameters		*Schistosoma* positive		
		β	[95% CI(β)]	*p* value
Erythrocytes (10^6^/mm^3^)		**0.006**	**[-0.09–0.10]**	**0.89**
	Haemoglobin (g/dl)	− 0.20	[− 0.43–0.02]	0.08
	Haematocrit (%)	− 0.61	[− 1.25–0.02]	0.06
	MCV (µm^3^)	− 1.50	[− 2.80 to − 0.21]	0.02
	MCH (pg)	− 0.54	[− 1.04to − 0.03]	0.04
	MCHC (g/dl)	− 0.02	[− 0.22–0.17]	0.83
Thrombocytes (10^3^/mm^3^)		**28.2**	**[10.1–46.4]**	**0.002**
Leukocytes (10^3^/mm^3^)		**1.13**	**[1.05–1.20]**	**0.0003**
	Lymphocytes (10^3^/mm^3^)	1.11	[1.04–1.18]	0.001
	Neutrophils (10^3^/mm^3^)	1.12	[1.03–1.22]	0.01
	Eosinophils (10^3^/mm^3^)	1.64	[1.36–1.98]	** < **0.001
	Basophiles (10^3^/mm^3^)	1.27	[1.13–1.43]	0.0001
	Monocytes (10^3^/mm^3^)	–	–	–

## Discussion

The main objective of the present analysis was to assess the effect of urogenital schistosomiasis on FBC parameters among schoolchildren living in Lambaréné, a semi-urban area. Our results reveal that children with schistosomiasis display an altered FBC profile with a change in the cell level count of the three cell types, as compared to those without the disease. However, taking into account some confounding factors which can either affect the haemoglobin and platelet levels [27] or schistosomiasis status such as PZQ treatment which is also known to normalise the leucocytes level in schistosomiasis infected children [4], the relationship between FBC parameters and schistosomiasis status observed was ambiguous for RBC; while, the observed change for thrombocyte and leukocyte counts was independently associated with the disease and was characterised by an increase in cell numbers.

Indeed, our results reveal a significant increase in total leukocyte count levels, as well as for each leukocyte type among children with schistosomiasis, as compared to those uninfected. The high WBC count levels we found among children with schistosomiasis, corroborate the reports of Mohammed et al. [4] and Afrifa et al. [10] among Sudanese and Ghanaian school children, respectively. We hypothesise that the high level of total leukocytes count observed is to be seen in connection with the host immune response against the presence of adult schistosomes in the bloodstream, and the particular role of eosinophils in the defense against helminthic infections is generally well recognised [28, 29]. Similarly, our results show a significant increase in thrombocyte count levels among *S. haematobium*-infected children. Based on what was demonstrated in brown rats [8], we hypothesise that thrombophilia is related to active defense mechanisms directed against adult worms.

With regard to erythrocytes; although the haemoglobin level was similar in both groups, the FBC yielded significantly lower haemoglobin and haematocrit levels among children infected with schistosomiasis, as compared to those non-infected. Similar erythrocyte profiles were recently reported by Afrifa et al. among Ghanaian children with urogenital schistosomiasis compared to those without [10]. In our study, however, when adjusting for history of PZQ intake, STH and *Plasmodium* spp. infection status, only a trend towards lower haemoglobin and haematocrit levels was observed; while, lower MCV and MCH levels remain significant. Indeed, the haemoglobin levels observed in this population were similar to the levels observed in children without the disease, and were above 11 g/dL, the threshold set by the WHO to define anaemia for populations living at sea level [30]. The absence of a statistically relationship between anaemia and schistosomiasis might appear surprising, since the main symptom of urogenital schistosomiasis is hematuria occurring by blood spilling during egg excretion. However, longer intervals between blood losses, few or no macrohematuria episodes and haematological recovery between episodes might be explanatory. *Plasmodium* spp. infection is usually associated with lower hematocrit and haemoglobin concentrations [31], and could explain the lower level of haemoglobin display by children with schistosomiasis. We indeed found an association between both infections. Interpretation of haemoglobin and platelets levels for one of these two diseases should, therefore, consider the possibility of the presence of both. Similar explanations could apply for cases with STH co-infections.

Some factors such as schistosomiasis chronicity and intensity could influence the relationship between schistosomiasis and FBC parameters we reported here and were not take into account in our analysis, particularly due to the study design. However, the large sample size included in this study should be reassuring with regard to the accuracy of our findings. The study was conducted among schoolchildren knowing to be most affected by the disease and our conclusions are limited to this population. However, further investigations should be conducted particularly among women also known to bear a high burden of urogenital schistosomiasis.

In conclusion, in our setting, schistosomiasis is associated with a characteristic FBC profile, indicating the necessity to consider schistosomiasis as a single cause of disease, or a co-morbidity, when interpreting an FBC in schistosomiasis-endemic areas.
